# Adult Intussusception due to Gastrointestinal Stromal Tumor: A Rare Case Report, Comprehensive Literature Review, and Diagnostic Challenges in Low-Resource Countries

**DOI:** 10.1155/2018/1395230

**Published:** 2018-08-06

**Authors:** Paddy Ssentongo, Mark Egan, Temitope E. Arkorful, Theodore Dorvlo, Oneka Scott, John S. Oh, Forster Amponsah-Manu

**Affiliations:** ^1^Center for Neural Engineering, Department of Engineering, Science and Mechanics, Pennsylvania State University, University Park, PA, USA; ^2^Department of Pathology, Eastern Regional Hospital, P.O. Box 201, Koforidua, Ghana; ^3^Department of Surgery, Eastern Regional Hospital, P.O. Box 201, Koforidua, Ghana; ^4^Ministry of Public Health, 1 Brickdam, Georgetown, Guyana; ^5^Department of Surgery, Penn State Hershey College of Medicine and Milton S. Hershey Medical Center, Hershey, PA, USA

## Abstract

We present a rare case of gastrogastric intussusception due to gastrointestinal stromal tumor (GIST) and the largest comprehensive literature review of published case reports on gastrointestinal (GI) intussusception due to GIST in the past three decades. We found that the common presenting symptoms were features of gastrointestinal obstruction and melena. We highlight the diagnostic challenges faced in low-resource countries. Our findings emphasize the importance of early clinical diagnosis in low-resource settings in order to guide timely management. In addition, histological analysis of the tumor for macroscopic and microscopic characteristics including mitotic index and c-Kit/CD117 status should be obtained to guide adjuvant therapy with imatinib mesylate. Periodic follow-up to access tumor recurrence is fundamental and should be the standard of care.

## 1. Introduction

Intussusception is the telescoping or invagination of the proximal part of the gastrointestinal tract (intussusceptum) into an adjacent section (intussuscipiens). Intussusception mostly occurs in childhood and is rare in adults with the incidence of approximately 2-3 per 1,000,000 per year, causing only 1% of all bowel obstruction in adults [1, 2]. Unlike the presentation of pediatric intussusception, in adults, the presentation is variable. Symptoms may be acute or chronic [3–5].

Furthermore, unlike intussusception in children where approximately 90% of cases are idiopathic, approximately 70%–90% of cases of adult intussusception are secondary to an underlying pathology, with 65% being due to benign or malignant neoplasms including GIST [1, 4, 6].

GISTs are mesenchymal tumors found in the GI tract possessing a range of malignant potential. They originate from neoplastic transformation of the interstitial cells of Cajal [7–10]. Although they can be found at any location along the GI tract, they frequently arise from the stomach or small intestines [10]. Their dynamic of growth being exophytic, they have a potential to invade the adjacent organs, and in some cases cause perforation into the peritoneal cavity [7]. With such pattern of growth, they rarely cause intussusception or obstruction. Here, we present a rare case of gastrogastric intussusception due to GIST in an 85-year-old woman and discuss diagnostic challenges and management in the low-resource environment. We also review 18 published cases of intussusception caused by GIST.

## 2. Methods

We present a rare case of gastrogastric intussusception due to GIST and a literature review of published studies on GI intussusception due to GIST. Searches were performed in the PubMed Central and Google Scholar databases. Keywords used were gastrointestinal stromal tumor, adult intussusception and intussusception caused by gastrointestinal stromal tumor, and GIST presenting as intussusception. The citations received via Google Scholar and PubMed Central were each further examined to determine if they satisfy the inclusion criteria. The database search included all articles from 1983 to February 2018. We extracted the following clinical characteristics: publication year, country of origin, patient age, sex, clinical history, duration of complaint, presence of palpable mass, imaging tools, surgical approach, tumor location, tumor size (largest dimension), CD117 expression, tumor mitotic index, length of follow-up after surgery, and recurrence status. If pertinent information was missing, corresponding authors were contacted with a list of variables to provide. We excluded articles of adult intussusception due to GIST that failed to report immunohistochemical staining of CD117 to confirm GIST.

## 3. Case Report

An 85-year-old Ghanaian female patient presented to our emergency department referred from a district hospital in Ghana with a 1-day history of melena associated with epigastric pain following food ingestion, dyspepsia, dizziness, and palpitations. The patient denied any history of hematemesis associated with this pain. The reason for referral from the district hospital was for a blood transfusion due to severe anemia. Prior to this, she also had a 14-day history of postprandial nausea and nonbloody vomiting. Physical examination revealed severe conjunctival pallor and melenic stool on digital rectal examination with a blood pressure = 110/70 mmHg, heart rate = 114 beats per minute, and afebrile temperature = 36.1°C. There was no abdominal tenderness or distention and no palpable abdominal mass on physical exam. Laboratory investigations showed macrocytic anemia (hemoglobin, 4.4 g/dL (normal: 12.3–18 g/dL), a hematocrit of 12% (normal: 40–54%), mean cell volume of 104.8 fL (normal: 80–100 fL), mean cell hemoglobin 53.5 pg (normal: 27–33 pg), and red blood cell distribution width 17.2% (normal: 11.0–16.0%)). Blood cell counts revealed a leukocytosis of 19,350/*μ*L (normal: 2600–8500/*μ*L), a neutrophilia of 14,570/*μ*L (normal: 2500–7500/*μ*L), and a platelet count of 392,000/*μ*L (normal: 150,000–400,000/*μ*L). The patient was resuscitated with 4 units of whole blood, normal saline, and ringers lactate. The differential diagnosis was upper GI bleeding secondary to peptic ulcer disease. The patient was started empirically on esomeprazole and had a nasogastric tube inserted. The patient continued to pass melenic stools and sustained severe anemia requiring continued blood transfusion. Due to the lack of resources including endoscopy, a functional computed tomography (CT) imaging unit, and inability to refer the patient 2 hours away to obtain imaging diagnostics, a clinical diagnosis of upper gastrointestinal bleeding was made based on the presence of melena and severe anemia, contrary to lower GI bleeding which usually presents with hematochezia. A decision for an emergent explorative laparotomy was done. Because this is a low-resource setting, there was no availability of endoscopy for laparoscopic surgery.

**Figure 1:**
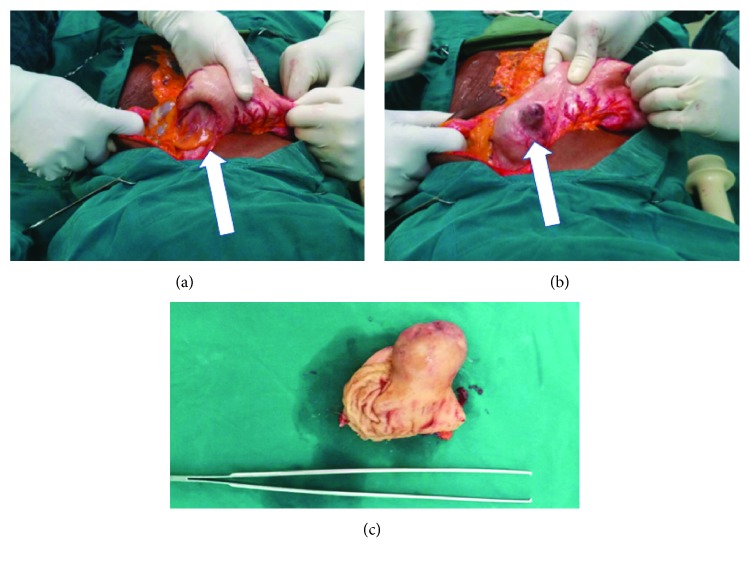
Intraoperative photograph of gastrogastric intussusception. (a) The fundus intussuscepting into the body of the stomach (white arrow). (b) GIST after reduction of the intussusception. The GIST is extending exophytically (white arrow). (c) A 2.5 cm × 2.5 cm excised GIST.

**Figure 2:**
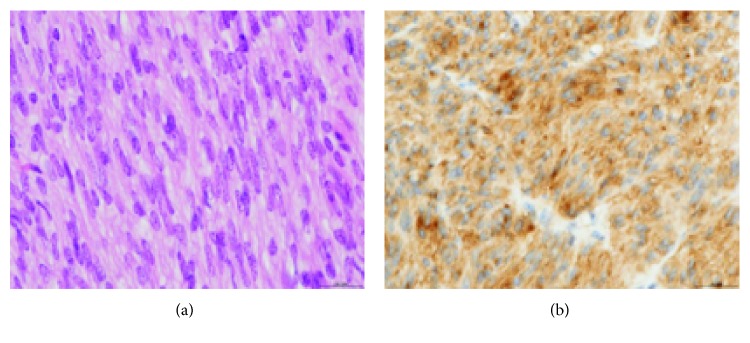
GIST histology. (a) H&E staining demonstrating spindle cells ×400. (b) IHC staining showing CD117 positive cells ×400.

Under general anesthesia, the abdominal cavity was entered through an upper midline incision. A gastrogastric intussusception was found. The gastric fundus was intussuscepting into the body of the stomach (Figure 1(a)). A tumor measuring 2.5 cm × 2.5 cm was found at the anterior fundal area (Figure 1(b)). The portion of the stomach at the level of the tumor was devascularized. The intussusception was reduced by gently applying pressure on the body of the stomach to reduce the intussusception. Wedge resection was performed at the fundus followed by primary anastomosis. The resected segment of the stomach measured 10 cm × 4 cm and weighed 0.2 kg. Macroscopic examination showed a cream to dark brown soft tissue mass. The tumor was completely resected with at least 0.2 cm clearance (Figure 1(c)). The hematoxylin and eosin staining (H&E) showed spindle cell in the muscularis of the stomach (Figure 2(a)). On immunohistochemical analysis, the spindle cells were positive for both c-Kit protein (CD117) and CD34 but negative for smooth muscle actin and desmin (Figure 2(b)). There were less than 5 mitoses per 50 high-power fields. A diagnosis of a low-risk gastrointestinal stromal tumor of the stomach was made. The patient recovered without complications, discharged 10 days later, and has remained well and symptom-free 2 years after discharge. She was not started on imatinib mesylate due to the small size and low mitotic index of the tumor.

### 3.1. Literature Review

We identified 28 reports concerning 28 cases of intussusception due to GIST. We excluded 10 reports because they failed to report immunohistochemical (IHC) staining for CD117 or failed to report the results of the analysis discovered on GIST-1 (DOG-1) or platelet-derived growth factor receptor alpha (PDGFRA) markers for the CD117-negative tumors. Therefore, we only included 18 reports concerning 18 cases of intussusception due to GIST in the literature review. The patients were aged 34 to 95 years (mean, 60 ± 15.8 years); 72% (*n* = 13) were women. 56% (*n* = 10) of GISTs were located in the stomach, 22% (*n* = 4) in the jejunum, 17% (*n* = 3) in the ileum, and 6% (*n* = 1) in the duodenum. 94% (*n* = 17) were CD117-positive, and 6% (*n* = 1) were CD117-negative. In 73% of the patients, there was no palpable mass on abdominal examination. The tumor dimensions ranged from 2.2 to 15 cm (mean, 6.2 ± 3.7 cm), and the median follow-up period was 12 months (range 3–33 months). There were no tumor recurrences reported. Regarding the types of intussusception, 56% (*n* = 10) of the cases were gastroduodenal, 17% (*n* = 3) were jejunojejunal, and 17% (*n* = 3) were ileoileal. Ileojejunal and duodenal-jejunal each contributed 6% (*n* = 1). None was gastrogastric. The clinicopathological characteristics of the 18 patients are summarized in Table 1.

## 4. Discussion

GISTs may occur anywhere along the GI tract with 60–70% of tumors occurring in the stomach and 20–25% in the small bowel [11]. This is in agreement with our findings in the literature analysis. In 1983, Mazur and Clark proposed the name stromal tumor to differentiate it from other smooth muscle gastrointestinal tumors [12]. The proposed cellular origin of GISTs are the interstitial cells of Cajal, intestinal pacemaker cells that regulate autonomous contraction of the GI tract [13]. Publications by two different groups in 1998 showed that GISTs commonly express CD117 and CD34 that are morphologically and immunophenotypically similar to the interstitial cells of Cajal [14, 15].

GISTs are one of the most common sarcomatous tumors of the gastrointestinal tract, with an incidence rate of 6 to 14 cases per million people in the United States of America and Europe [16] and approximately 16 to 22 cases per million people in Asia [17]. The incidence in Africa is unknown. The incidence rose as a result of the introduction of anti-CD117 antibody for immunohistochemical staining in 2001. This was due to the change in diagnostic methods and to the reclassification of many mesenchymal gastrointestinal tumors previously diagnosed as smooth muscle tumors such as leiomyosarcomas [18].

A review of 18 cases of intussusception secondary to GIST found that approximately 56% of GISTs were located in the stomach followed by a quarter of tumors arising from the jejunum. We also found that over half of the types of intussusception were gastroduodenal. Mucosal ulceration or fistulation occurs in about 15–50% of these tumors. The associated bleeding in our patient likely contributed to her anemia. Pathohistologically, GISTs are defined by positive immunostaining for c-Kit protooncogene-CD117 (overexpressed in 95%) and CD34 (positive in 60% to 70%) [19].

GISTs most commonly present with dyspepsia and GI bleeding presenting as melena caused by pressure necrosis and ulceration of the overlying mucosa [20]. Rarely, they may present with bowel obstruction or tumor rupture with hemoperitoneum. In our study, 28% of patients presented with melena and 83% presented with vomiting. The classic triad of intussusception, abdominal tenderness, palpable abdominal mass, and hemoglobin-positive stools, is rarely found in adults [21]. Therefore, an accurate diagnosis is based on a combination of accurate medical history, thorough physical examination, and imaging modalities.

Abdominal X-ray is the first diagnostic tool used due to the obstructive symptoms that dominate the clinical picture in most cases. However, due to its high sensitivity (98–100%), specificity (88%), and a lower cost, abdominal ultrasound scan (US) is the diagnostic tool of choice [22]. The typical imaging features of abdominal US consist of the doughnut or target sign in the transverse view and the pseudokidney or sandwich sign in the longitudinal view. Barium studies in upper GI series show stacked coin or coiled spring sign due to edematous mucosal folds and a cup-shaped filling defect in barium enemas when evaluating colocolic or ileocolic intussusception [23]. However, due to the higher sensitivity of abdominal computed tomography (CT) scans [24] and the characteristic “target sign,” it has been reported to be the most useful and accurate imaging modality for diagnosis of intestinal intussusception and may be superior to the abovementioned studies.

In low-resource countries where access to imaging modalities like CT scan and endoscopy is a challenge [25], a timely diagnosis should be made based on a clinical history and physical examination. The clinical presentation includes abdominal pain, nausea and vomiting, and melena. The definitive diagnosis of intussusception is made intraoperatively due to the paucity of preoperative imaging. In light of the patient's massive bleeding, with no endoscopic capability and limited blood products, the decision to perform an exploratory laparotomy for hemorrhage control was made. If the laparotomy was not done urgently, the patient would have died due to severe anemia. In our case, we performed a laparotomy on the grounds of clinical findings and in the absence of access to imaging means such as an abdominal US and CT scan or a plain X-ray. In this environment, any delay in surgery resulting in necrotic bowel complicates management and may necessitate an otherwise avoidable bowel resection. The resulting complications may include the need for an ostomy, anastomotic leak, and reoperation. All of these complications further burden the healthcare system in an economically overstressed system.

Treatment of adult intussusception is always surgical [26]. However, optimal management remains controversial. The surgical approach is either primary *en* block resection or initial reduction of the intussusception followed by a limited resection [27]. However, suspicion of malignancy is a contraindication to reduction to avoid the likelihood of intraluminal seeding, venous embolization in regions of ulcerated mucosa, and anastomotic leak [28]. Laparoscopy as a minimally invasive procedure for both diagnosis and treatment of adult intussusceptions has recently gained popularity [29]. For surgical resection of a gastric GIST, a laparoscopic approach is associated with low morbidity, mortality, and short length of stay, and therefore, if available, is the preferred resection technique in the majority of patients having small- and medium-sized gastric GISTs [30]. In addition to surgical management of GIST, imatinib mesylate is used if the tumor is aggressive. This drug was approved by the FDA in 2001 for the treatment of gastrointestinal stromal tumors. Its mechanism of action is to selectively inhibit the KIT signal-transduction pathway (the mutated exon 11 of the KIT receptor) [31]. Patient age, tumor size, mitotic index, tumor ulceration, and necrosis significantly influence tumor recurrence. However, the presence of 10 or more mitotic figures per 50 high-power fields is an independent and a significant predictor of disease progression [30]. The 2-year survival of patients with advanced disease has risen to 75–80% following treatment with imatinib mesylate. In our literature review, we found that approximately 28% of the patients were started on imatinib mesylate after surgery. There was no tumor recurrence reported in the median 12 months of follow-up.

## 5. Conclusion

Although gastric GIST is not uncommon, presentation in the form of gastrogastric intussusception is very rare. This diagnosis should be entertained in a patient with acute gastric outlet obstruction and melena. In low-resource countries with limited access to imaging modalities, clinical history and physical exams should be the basis of early diagnosis and surgical management. Surgical management is the best treatment modality. After reduction of the intussusception, GIST requires surgical resection and should be histologically analyzed to quantify its aggressiveness.

## Figures and Tables

**Table 1:** General characteristics of 18 cases of intussusception due to gastrointestinal stromal tumor reported between 1983 and 2018.

Reference	Country	Age (year)	Gender	Presentation	Duration of complaints	Palpable mass	Imaging tool	Surgical approach	Tumor location	Tumor size largest dimension (cm)	Expression for c-Kit/CD117, mitotic index	Follow-up/recurrence
[32]	Greece	79	F	Lower right abdominal colicky pain, abdominal distention, N + V	5 days	No	Plain X-ray, contrast CT	Laparotomy, end-to-end ileoileal anastomosis	Ileum	2.2	Positive, 7-8 mitoses/50 HPF	11 months, no recurrence
[33]	Brunei	62	F	Epigastric pain, melena	3 days	No	Endoscopy, CT	Billroth II, partial gastrectomy	Distal body of the stomach	5.2	Positive, 6 mitoses/50 HPF	Taking imatinib mesylate, no recurrence
[34]	China	34	F	Epigastric pain, vomiting	1 month	No	CT, endoscopy	Laparoscopic, wedge resection	Fundus	6.5	Positive, 2 mitoses/50 HPF	No recurrence, on follow-up
[35]	USA	52	F	Epigastric pain, vomiting	1 day	No	CT, endoscopy	Laparoscopic, wedge resection	Fundus	5.0	Positive, 4 mitoses/50 HPF	5 months, no recurrence, taking imatinib mesylate
[36]	Japan	95	F	Vomiting and loss of appetite, melena	1 week	NR	CT, endoscopy	Endoscopic submucosal dissection	Posterior wall of distal body	4.2	Positive, 4 mitoses/50 HPF	No recurrence, patient died of old age 55 months later
[37]	Japan	51	M	N + V, melena, and severe anemia	4 days	No	CT, endoscopy		Antrum	5.5	Positive/NR	No recurrence
[38]	India	65	F	Upper abdominal pain, intermittent vomiting 30 minutes after meals	6 months	Yes	CT, endoscopy	Laparotomy, wedge resection	Pylorus	6.0	Positive, 0-1 mitosis/50 HPF	1 year, no recurrence
[39]	Ireland	78	F	Upper abdominal discomfort, vomiting, and anorexia	1 week	NR	CT	Endoscopic reduction, laparoscopic, wedge resection	Body and antrum	4.5	Positive, NR	No recurrence on follow-up
[40]	Ghana	59	F	Intermittent vomiting	1 week	Yes	US	Laparotomy, wedge resection	Anterior wall stomach	NR	Positive, <1 mitosis/50 HPF	12 months, no recurrence
[41]	India	60	F	Intermittent vomiting 30 minutes after meals, loss of appetite and weight	NR	NR	CT, endoscopy	Laparoscopic, Billroth II, partial gastrectomy	Antrum	8.0	Positive, 2 mitoses/50 HPF	14 months, no recurrence
[42]	China	69	F	Acute abdominal pain, N + V	6 hours	No	Endoscopy	Laparoscopic wedge resection	Antrum	4.5	Negative, but DOG-1 and CD34-positive, no PDGFRA mutation, < 5 mitoses/50 HPF	33 months, no recurrence
[43]	UK	68	M	Abdominal pain and distension, vomiting, constipation, melena	NR	NR	CT	Laparotomy	Jejunum	4.0	Positive, 0-1 mitosis/50 HPF	NR, no recurrence
[44]	UK	70	M	Abdominal pain, nausea, bilious vomiting, constipation	1 week	NR	Abdominal X-ray, CT	Laparotomy, primary anastomosis	Jejunum	4.0	Positive/NR	3 months, no recurrence, taking imatinib mesylate
[45]	Morocco	59	F	Abdominal distension, pain, constipation, vomiting	6 months	No	CT	Laparotomy, primary ileoileal anastomosis	Ileum	NR	Positive/NR	NR
[46]	India	46	F	Abdominal pain, abdominal distension, anorexia, vomiting, constipation	36 hours	Yes	Endoscopy, US, CT	Laparotomy, primary jejunojejunal anastomosis	Jejunum	4.0	Positive, 6 mitoses/50 HPF	2 years, no recurrence, taking imatinib mesylate
[47]	India	38	M	Abdominal pain	2 months	Yes	US, CT enteroclysis	Laparotomy, tumor resection	Jejunum	15.0	Positive, 6 mitoses/50 HPF	Six months, no recurrence, taking imatinib mesylate
[48]	India	59	M	Abdominal pain, distension, bilious vomiting, constipation	3 days	No	Plain X-ray abdomen, US	Laparotomy, primary ileoileal anastomosis	Ileum	NR	Positive/NR	NR
[49]	UK	36	F	Collapse, melena, hypotension (82/46 mmHg), tachycardia (150 bpm)	NR	No	CT	Laparotomy, pancreaticoduodenectomy	Duodenum	15.0	Positive/NR	No recurrence

F: female; M: male; NR: not reported; US: ultrasonography; CT: computed tomography; N + V: nausea and vomiting; HPF: high-power field; USA: United States of America; UK: United Kingdom; bpm: beats per minute; DOG-1: discovered on GIST-1; PDGFRA: platelet-derived growth factor receptor alpha.

## Additional Points

*SCARE Checklist (2013) Statement.* The authors have read the CARE Checklist (2013), and the manuscript was prepared according to the CARE Checklist (2013).
